# In Silico Predictions on the Productive Life Span and Theory of Its Developmental Origin in Dairy Cows

**DOI:** 10.3390/ani12060684

**Published:** 2022-03-09

**Authors:** Evgeniy Kharitonov, Gennadiy Cherepanov, Konstantin Ostrenko

**Affiliations:** Russia Research Institute of Animal Physiology, Biochemistry, and Nutrition—Branch of the Federal Science Center for Animal Husbandry Named after Academy Member L. K. Ernst, 249010 Borovsk, Russia; 89611243110@mail.ru (G.C.); ostrenkoks@gmail.com (K.O.)

**Keywords:** dairy cows, viability, welfare, productive life span, heterogenous populations, computational modeling

## Abstract

**Simple Summary:**

Dairy cows are susceptible to a range of welfare factors, which lead to worsening health problems and shorten their productive life span. The health and welfare status of dairy cows could be improved if unwanted abnormalities and risk factors are detected in a timely manner, i.e., before diseases start to occur. Therefore, in addition to veterinary monitoring, quantitative parameters are necessary to predict the risks of early culling of cows. In the study of the age dynamics of culling rate in dairy cow populations, it was found that the average productive life span can be predicted by registration of the reciprocal relative disposal rate (culling for sum of reasons + death). This indicator represents the viability index, which has a maximal value at the first lactation and decreases in subsequent lactations with an inverse exponential trend. According to available scientific information, the structural prerequisites for this index are laid down during prenatal development and in the early periods of postnatal life; therefore, it is necessary to create a system of continuous monitoring of the physiological status of mothers and young animals.

**Abstract:**

Animal welfare includes health but also concerns the need for natural factors that contribute to the increase in viability. Therefore, quantitative parameters are necessary to predict the risks of early culling of cows. In the study of the age dynamics of the disposal rate (culling for sum of reasons + death) in dairy cow populations, it was found that the average productive life span can be predicted by the value of the reciprocal culling/death rate (reciprocal value of Gompertz function) at the first lactation. This means that this potential of viability is formed during the developmental periods preceding the onset of lactation activity. Therefore, taking into account current data in the field of developmental biology, it can be assumed that the structural prerequisites for viability potential are laid down during prenatal development and in the early periods of postnatal life. To prevent unfavorable deviations in these processes due to negative welfare effects, it is advisable to monitor the physiological status of mothers and young animals using biosensors and Big Data systems.

## 1. Introduction

Dairy cows are susceptible to a range of negative welfare factors, such as stall discomfort, metabolic and physical stress, bad pasture condition, age, parity, etc., which in general lead to worsening health problems and shorten their productive life span [1,2,3]. Animal welfare includes health but also concerns the need for natural factors (e.g., access to pasture) that contribute to the increase in viability [4,5]. Risks associated with a higher culling rate include increasing herd size, average milk yield and morbidity, respiratory disease, and total mixed ration feeding [6,7]. An increase in milk production has been associated with risks of altering behavioral, physiological, and immunological conditions [1], leading to greater risks for health disorders, which are the primary reported reasons for culling. On the other hand, improvements in cow comfort, reproduction, and genetic merit for productive life in recent decades have not markedly led to increases in the productive lifespan of dairy cattle [5,8]. The most general quantitative indicator of welfare for dairy cow populations seems to be the length of productive life.

An increase in involuntary culling in the herd indicates poor animal health and inefficient use of animal resources, which oppose sustainable dairy production [9]. The health, welfare status, and length of productive life (PLS) of dairy cows could be improved if unwanted abnormalities and risk factors are detected in a timely manner, i.e., before diseases start to occur. Therefore, in addition to veterinary monitoring, procedures of prenosological diagnostics and quantitative parameters are necessary to predict the risks of early culling of cows. If we are unable to identify risk factors and measure the parameters necessary to predict viability, then we will not be able to control health and welfare status in the system of physiological monitoring.

Among the many physiological parameters of this type can be attributed indicators of the organism’s resistance to the effects of external and internal factors that reduce vitality, including indicators of immunity and nonspecific (innate) resistance, which in humans are determined according to data of a clinical blood test or analysis of tissue probes to confirm a suspected disease. With regard to the task of monitoring the health and welfare of cows, these indicators are unacceptable for mass examination; moreover, they are highly variable and not suitable for predictive veterinary medicine and production management. As an example of the possible options of prenosological diagnostics, periodic measurements of body condition score (BCS), daily milk yield, and milk composition are currently used with the aim to prevent metabolic stress, hypoglycemia, fatty hepatosis, and decreases in immunity in cows in the transit period of lactation [2,5,10,11,12].

As for the problem of cow longevity, it should be borne in mind that analytical apparatus has long been successfully used in studies of life span in human mortality [13]. It was originally proposed 200 years ago in the form of a differential equation describing the age dynamics of human mortality (Gompertz function). In gerontology, the main interest represents periods of old age, i.e., maximal longevity of humans, but dairy cows leave the herd at earlier age periods, and cow mortality, formally, is similar to the disposal rate of culling for the sum of natural reasons and death. Historically, Gompertz [13] was the first to suggest the concept of general constitutive resistance (CR), which he called resistance to mortality, as the reciprocal of the relative mortality rate.

Methodological approaches based on the use of various modifications of the Gompertz function are applicable in the analysis of the survival rate of laboratory animals and dairy cows [14,15,16,17,18,19,20,21,22,23,24]. The assumption is substantiated that in order to reduce the loss of productive animals from numerous polyetiological diseases (including so-called “productivity diseases”, i.e., diseases of the udder, reproductive organs, legs, etc.), it is necessary first of all to track and control the age-dependent decline in the total background viability (i.e., CR) of the organism. The novelty of this approach lies in the orientation toward the diagnosis and correction not of specific diseases, but to the earlier detection of hidden factors that predispose to a decrease in productive life span (PLS) as milk production increases [24,25].

The aim of this work was to solve two problems: (1) to develop a methodology for in silico forecasting the productive life span (PLS) in dairy cow populations and to test it on an empirical data basis; (2) to interpret the obtained new data in light of the hypothesis of the developmental origin of PLS variability in highly productive herds.

## 2. Materials and Methods

### 2.1. Study I. Analysis of Age Dynamics of Disposal Rate and In Silico PLS Forecasting

As a material for this study, data on the number of cows in subsequent lactations registered in 15 production units (breeding plants, individual districts) of Black-and-White cows in Leningrad oblast in the period 1985–2000 were used. An example of input data is presented in Table 1.

Paired data for 4–5 and subsequent lactations were reconstructed, and the corresponding values for individual lactations were restored by selecting corrective coefficients according to the criterion of the minimum sum of deviations from the general trend, predicted by approximating the resulting series with the Gompertz function *y*(*t*).

(1) $$y\left(t\right)=\frac{dN\left(t\right)}{dt\times N\left(t\right)}=B\times \exp(c\times t)$$

where *t* is the time variable in the form of the number of lactations, *N*(*t*) is the current size of the cohort, and *B* and *c* are constants. Parameter *c* is an indicator of aging rate, and *B* is the initial level of the relative disposal rate (d*N/N,* i.e., the value of relative culling for sum of involuntary reasons + death) at the first lactation.

The dynamics of the disposal rate of dairy cows can be assessed according to the data of the disposal rate in the cohort (a group of individuals of the same year of birth, i.e., a “longitudinal” method, or by data on the number of cows in a herd with sequential numbers of lactations (“cross” method). Successive groups in a herd are the remnants of preceding cohorts; therefore, these two methods give the same results under the preposition of constant patterns of disposal rate for all lactations in successive years. The difference from human studies of longevity is that for cows, there are relatively few age points (lactation numbers); therefore, multiparameter models, used in human gerontology, are not used here, and only two parameters can be used: (1) *c*, indicator of aging rate; and (2) *B,* initial level of disposal rate, which for cows is measured at the first lactation.

**Figure 1:**
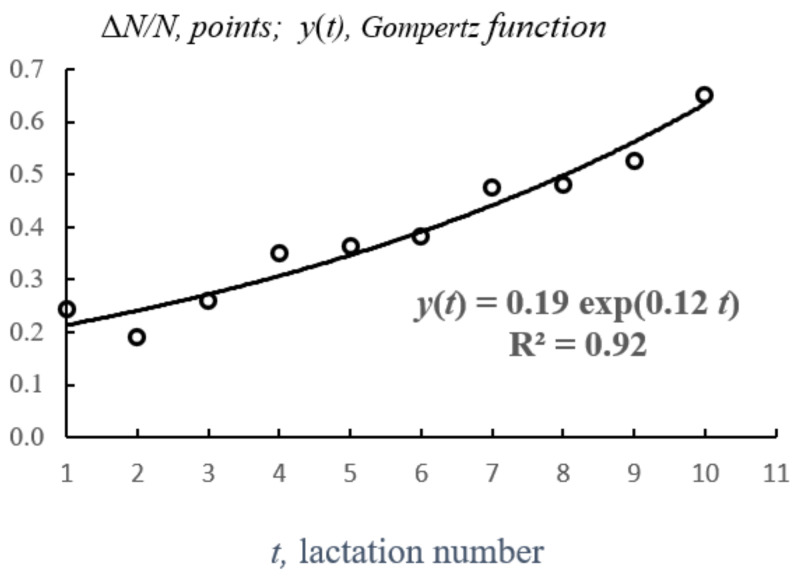
Empirical trend of the age-related increase in the disposal rate of the cows (Δ*N*/*N*) and the approximating Gompertz function *y*(*t*); *t* is lactation number.

To approximate a series of empirical (input) data by the Gompertz function and use it for PLS forecasting, a novel methodology was developed, in which the differentials *dN* and *dt* are replaced by unit intervals Δ*N* and Δ*t* under the condition Δ*t* = 1 (step along the time axis = 1, one lactation). In this case, using tables in Microsoft Excel, in the first column, the lactation numbers *t_i_* are set, the second column is Δ*Ni* (i.e., Δ*N1 = N2 − N1* for *t*_1_ = 1, and so on), and the third column is sequence Δ*Ni*/*Ni* (i.e., the sequential values of the Gompertz function). The sequence Δ*Ni*/*Ni* is used to estimate the values of *B* and *c* by specifying the form of the approximating function *y*(*t*) *= B* × exp (*c × t*) and building a point diagram with an indication of the exponential trend, parameters of the regression equation, and the value of R^2^ (Figure 1).

To solve Equation (1), i.e., to restore the sequences of *Ni* according to the given values of *B* and *c* in the Gompertz function *y*(*t*), in the first column, the lactation numbers *t_i_* (i.e., *x*) are set, and the sequential values of *y* = B × exp(*c × x*) are restored in the second column. In the third column, the values of (1 − y_i_) are set. In the first cell of the fourth column, the conditional value of the initial size of the cohort (*N*1 for first lactation, usually at least 1000–1500) is set. Insofar as *y1* = Δ*N1*/*N1* = (*N*1 − *N*2)/*N1*, and *y*1 × *N*1 = *N*1 − *N*2, *N*2 = (1 − *y*1)*N*1, and so on. This operation is repeated for all subsequent *t_i_* values until a negative *N*i value appears at the *i*-th iteration (the predicted maximum life span of cows in a given herd is determined by the *N*(*i*-1) value) (Table 2).

To forecast the productive life span of a population that is heterogeneous in terms of parameters *B, c,* and initial cohort size, sequences *Ni* for successive lactations can be built by setting different combinations of values *B, c,* and *N1* and summing these series. Thus, a model population is obtained that is heterogeneous in terms of parameters and initial cohort size. For the constructed sequence of *N*_sum i_, the parameters of the Gompertz function are found, and a point diagram of *y*_sum_(*t*) for the mixed population is constructed. The model diagram and empirical diagram can be compared with the aim of testing and verifying the hypothesis about the possible causes of the heterogeneity of the studied population (see Section 3).

### 2.2. Study II. Age Dynamics of 305 d Milk Yield in Cows with Various Values of PLS

Data on milk production were registered as 305 d milk yields in one herd of Kholmogor cows for all successive lactations in groups with different lengths of productive life, culled during several years (2002–2005, total number of cows *n* = 1500). The reasons for disposal/culling were not taken into account. The distribution curves of the relative frequency of first calving over the year for the 6 groups were similar (with some statistical scattering), i.e., there was an overall tendency toward a uniform distribution with local elevation during February–April. Additionally, the subsets of cows with a given number of lactations were distributed uniformly over a relatively broad range of years. Therefore, these data suggest that quantitative biases with year-seasonal effects were nonsignificant.

Material and methods. When analyzing the age-related dynamics of milk productivity, data on milk yields for 305 days in groups of cows with different lengths of productive life (with numbers of last lactation 4, 5, …, 10) were used. To assess the quantitative parameters that determine the age dynamics of 305 d milk yield for successive lactations (y*_m_*, kg; *t* is lactation number), a three-component regression function was used:

*y_m_* (*t*) = *A* × exp(−exp(−b*t*)) × D*^t^* (2)

where *A* is a constant parameter that has a potential value of 305 d for this group of cows. The product of A and the second component, exp(−exp(−b*t*)), describe the increase (with access to the plateau level at the 5–6th lactation) in the potential ability to produce milk due to an increase in body size and cytomorphological development of the udder. The numerical value of parameter *b*, according to preliminary estimates, varies within relatively small limits (0.4–0.5). Actual 305-day milk yields for successive lactations in each group were determined by the product of *A* × exp(−exp(−b*t*)) and the degradation component D*^t^* (D < 1), which describes the rate of age-related decline (D < 1) in the functional capacity of the milk production system, while the value of D is the “initial” value of this parameter for the first lactation (D^1^ = D). The value of D is determined by extrapolating the linear regression trend, built in the interval of 4–10 lactations, to the first lactation.

## 3. Results

### 3.1. Study I. Express Method of Using Gompertz Equation to Estimate Viability Indicators for Dairy Cow

Based on published input data for five US breeds , a linear relationship between 1/*y*1 and average productive life span *T* was found (r = 0.94, *p* < 0.05).

A similar relationship was found using data on 15 production subdivisions of Leningrad oblast (breeding plants, individual districts):

T = 0.26 (1/y1) + 1.2; R^2^ = 0.99, *p* < 0.001). (3)

The data obtained indicate that the length of productive life significantly depends on the value 1/*y*1 = *B^−1^*×,72 = *N*1/ΔN1 as a parameter characterizing the viability potential (initial value of viability indicator), which was formed before the onset of lactation activity.

**Figure 2:**
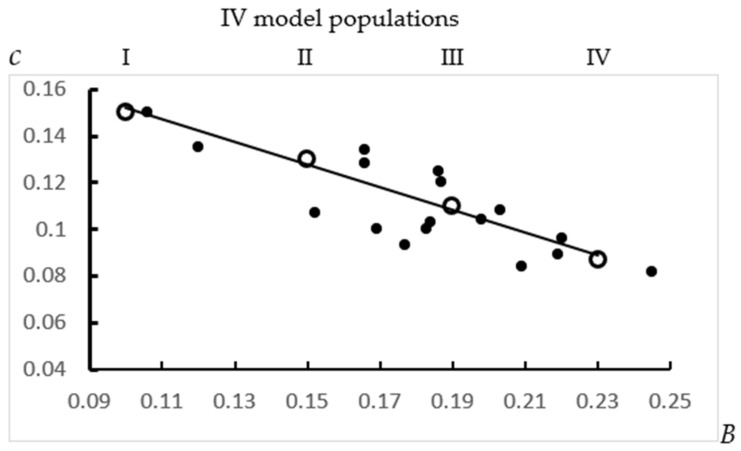
Correlation between the values of parameters *B* and *c,* found in the analysis of cow culling rate in 15 production units of Leningrad oblast (total size of whole population ≈ 35,000 dairy cows). ●—empirical data; ○—model forecast for IV model populations with the same values of *c* = 0.15; I is homogenous, II consists of two, III and IV consist of three subpopulations, with different values of *B* and *N*1 (initial number of cows in cohort) (Table 3).

In a study performed in 15 production units on Black-and-White cows in Leningrad oblast (breeding plants, individual districts), a correlation was found between the values of parameters *B* and *c* (Figure 2). To explain the revealed correlation, the assumption was investigated that if all 15 populations were homogeneous by parameter *c*, but some of them (or most of them) were heterogeneous by parameter *B*, this would lead to a decrease in parameter *c* for all heterogeneous populations. The results of a series of calculations confirmed the correctness of this assumption, since the regression line constructed from empirical data coincided with the trend line constructed from model data for four heterogeneous populations (the input data for this forecast are given in Table 1).

Thus, in the process of preliminary verification of the concept, carried out on the data of the registration of the age dynamics of cows culling, it was found that the average PLS in the dairy cow population can be predicted by the initial value of the reverse relative culling of cows at the first lactation. In other words, as the predictor of PLS, the magnitude of a trait that was formed during the development periods preceding the onset of lactation activity can be used.

When considering the scatter plot shown in Figure 2, the location of the leftmost point, indicating the homogeneity of this population, can be interpreted with sufficient reliability in the sense that this population has no health and welfare problems, while on the right side of the diagram, the short-lived subpopulations appear with reduced welfare status.

In this work, the ability to predict PLS was established on the basis of population data, but a similar test, in principle, can be developed at the individual level [12]. For such complex and highly variable traits, such as viability and longevity, conclusions based on population data are of primary importance, although practical application will undoubtedly require more detailed studies, taking into account the specific conditions of dairy farm conditions.

### 3.2. Study II. Age Dynamics of 305 d Milk Yield in Cows with Various Productive Life Span

**Figure 3:**
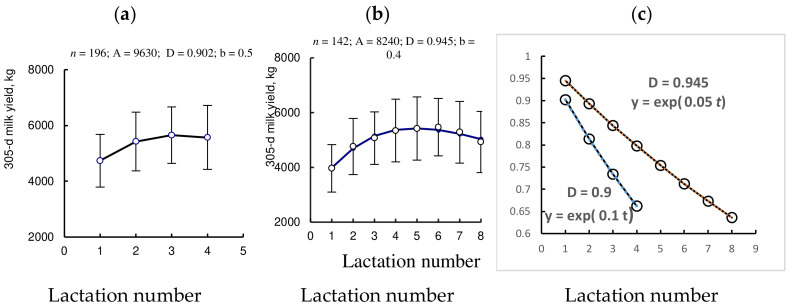
Age dynamics of 305 d milk yield (**a**,**b**) and degradation component D*^t^* (**c**).

The initial value of the degradation component, D in Equation (2), can be interpreted as the potential of protective forces (viability, general background resistance, etc.) formed before the age of reproductive maturity. It can vary from 0.85–0.87 in cows with a short productive life to 0.99 in long-lived cows (Figure 3). With this interpretation, the dynamics of indicator D*^t^* over long periods of time represent the dynamics of the general background resistance (including innate immune resistance) to the action of factors associated with lactation activity and age, while the overall actual resistance in certain short periods of time can be made up of background constitutive and inducible components (including reactions of humoral and cellular immunity for antigen intrusion).

In the analysis of the interrelationship between indicators D and *t_max_* (number of last lactation) for seven groups, a regression equation was obtained:

D = 0.88 + 0.008 *t*_max_, r = 0.94, *p* < 0.001, i.e., *t*_max_ = 125 D − 110 (4)

In another study conducted on a smaller population of White-and-Black cows (*n* = 195), a similar linear relationship was obtained: D = 0.88 + 0.012 *t _max_*, r = 0.84, *p* < 0.001; i.e., *t*_max_ = 83 D − 73.

**Figure 4:**
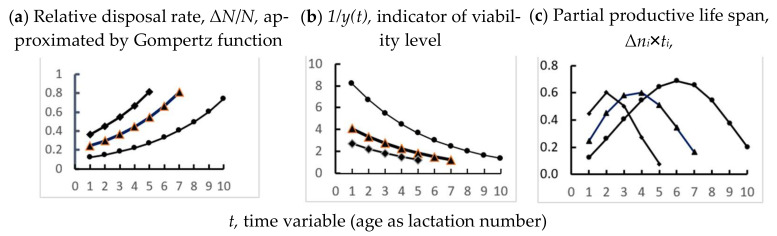
Age dynamics of three indicators of herd turnover for 3 model cohorts. *y*(*t*) is Gompertz function as relative culling (Δ*Ni*/*Ni*); 1/y(t) is indicator of viability of cows; Δ*n_i_* = Δ*N*i*/N*1, *t_i_*—lactation number, Δ*n_i_*×*t_i_—p*artial productive life span, i.e., relative number of cows in herd with productive life span equal to given lactation number. The average productive life span *T* = Σ Δ*n_i_*×*t_i_*. For all 3 variants: *c* = 0.2; ● B = 0.1; ▲B = 0.2; ♦B = 0.3.

When comparing Equations (2) and (4), an analogous relationship can be seen between the PLS and the values of viability indicators obtained by analyzing the age dynamics of culling rate and 305-day milk yield in the studied populations of cows. In addition, comparing Figure 3c and Figure 4b (see Section 4) reveals similar patterns in the position of the initial viability level, 1/y(t) and indicator D, i.e., the higher the initial position of the viability indicator, the longer the PLS of the dairy cows.

## 4. Discussion

Based on the analysis of the results obtained using different methodological approaches in Studies I and II, it can be concluded that the observed variations in PLS in the studied dairy cow populations mainly depend on the parameters characterizing the initial level of viability that was formed in the periods preceding the onset of lactation activity (1/(y1) in Study I and D in Study II). At first glance, the value of this fact is that it can be used to predict the PLS of cows, but this is not entirely true. Firstly, when analyzing the data of Study II on the age-related dynamics of milk yield, the initial value of D is not measured, and its relationship with the value of PLS is revealed by analysis of six subsequent lactations. Secondly, the high statistical significance of the relationship between the mean PLS and the value of 1/(y1) in Study I has so far been established only for a specific large population, and for the practical application of such a test, one study is insufficient. In addition, for small populations, the performance of this test will be smaller. Finally, the usefulness of predicting longevity is doubtful if the possible causes and mechanisms underlying early culling of cows are not known.

One of the essential elements of novelty in the results of this work can be expressed as a new look at an old problem. The novelty here is that a methodology for measuring viability was proposed, accessible to a wide range of livestock specialists who do not have a thorough mathematical background. This is important due to the fact that these biological traits are extremely variable, so the study must be carried out on large populations with the fixation of numerous production factors. Practice shows that hoping for success along the path of establishing joint work with professional mathematicians in such situations is nonperspective (“mathematicians do not know biology, and biologists are not strong in higher mathematics”). The described sequence of simple arithmetic operations can be called a computational experiment (in silico), which can be carried out to test the hypothesis about the heterogeneity of the studied population in terms of parameters of survival. There is no reason to consider this procedure as mathematical modeling, since no new models are built, the Gompertz law is used, and sequences of arithmetic operations are made in order to identify factors that modify the manifestation of this general law in specific conditions.

In the same way, when creating new aircraft, engineers carry out calculations using previously established laws of aerodynamics. Law may be created once a century, but the real effect is achieved by the work of numerous engineers who solve specific problems. The Gompertz equation is not an empirical regression equation; it is a fundamental law, and, like any law, it is carried out always and everywhere “in its pure form” under certain conditions. For the Gompertz law, these are the conditions for the homogeneity of the cohort and the stationarity of the renewal of the population (the constancy of the percentage composition of the dairy herd for successive years). Therefore, only an armada of zoo engineers is capable of solving the problems of viability and survival, taking into account the vast variety of biological and production factors. A small number of points is not a big drawback when analyzing the survival rate of cows, having a short life span, since first lactations are characterized by the highest rate of decline in viability, while intergroup differences are more clearly manifested.

According to preliminary data, aging parameter *c* in homogeneous cohorts, probably, is determined by hereditary factors; for example, it may be breed specific, and the initial level of culling can be determined to a large extent by the epigenome, i.e., epigenetic modifications in the processes of prenatal development and in the early periods of postnatal life. On the other hand, if the studied population is heterogeneous by survival parameters, the empirical Gompertz function (for example, Figure 1) is, in essence, of little information. Moreover, without taking into account the heterogeneity factor, the information content of some physiological and breeding tests may be lost to some degree. Some effects of heterogeneity are illustrated in Figure 4, when comparing the age trends of parameters measured by the proposed method (Δ*N*/*N, 1/y(t), Δn_i_*×*t_i_*) in a model herd that is heterogeneous by parameter B in the Gompertz function. The shortest-lived group disappears at the fifth lactation, and at the seventh lactation, only one long-lived subpopulation remains in the herd, although in this situation, parameter *c*, determined by hereditary factors, has the same value for these three groups. If physiological and biochemical studies are carried out on cows of the third or fourth lactations, the studied samples will be represented by individuals with different survival potential and, accordingly, with different functional and metabolic parameters.

The second significant element of novelty in the results of this study can be considered the establishment of the fact that the correlation between parameters *B* and *c* in the Gompertz function can be due to the heterogeneity of the studied populations by parameter B at the same value of *c*. This correlation, known since the middle of the last century as the Strehler–Mildwan correlation, still does not have an unambiguous explanation in the literary sources in the field of demography and gerontology. On the other hand, the establishment of this fact can be regarded as an additional argument in favor of the developed concept of the role of the level of “initial” viability for PLS of cows.

A second new look at the old problem of the viability of high-yielding cows in this paper is an attempt to shift the focus from disease control to the study of factors predisposing to the occurrence of prediseases and risk factors, which, with a high degree of probability, can be found at stages far removed from the onset of clinical symptoms of diseases.

Currently, there are many experimental and theoretical studies describing the functioning of physiological homeostasis systems (operating in “fast” time), but the number of works that consider the behavior of biological systems in “slow” time is much less. The object of such studies is usually not homeostasis, but homeoresis—a continuous series of homeostasis, the trajectory of changes in the state of the system over time. This corresponds to that proposed in the 1960s of the last century by Waddington’s model of ontogenesis as a creod. Unlike homeostasis as a return to one specific point of the initial state after a forced deviation, in the creod, model this point does not remain stationary but drifts in time, and the trajectories described by this phase point in the process of compensating for deviations seem to be attracted to the central, canalized trajectory, which is the creod.

Visually, the creod model can be represented as an inclined trough, along which a ball rolls, experiencing lateral shocks, tending to push it over the side (as in bobsleigh track). The higher the height of the upper base of the trough, the longer the duration of the descent (similar to life span). Within the framework of this model, the compensation of all variations of the ball trajectory caused by lateral shocks should be attributed to the effects of the adaptive (inducible) resistance component, and movement along the center of the trough should be attributed to the background resistance. The second component is difficult to assess in a short-term experiment, but, in all likelihood, it determines the long-term effects of animal general background resistance and viability.

This is indicated by the relationships between the productive life span and the “initial” level of parameters, which we revealed in the analysis of the age dynamics of 205 d milk yield and disposal rate in dairy cows (Figure 3c and Figure 4b). It is important to keep in mind that at each stage of ontogenesis, the systems of physiological homeostasis function, in principle, in the same way and differ only in the efficiency of regulation, which gradually decreases with age. In other words, the general background (constitutive) resistance is “masked” by the uniformity of manifestations of inducible adaptive resistance at all stages of ontogenesis.

All multicellular organisms, including insects, laboratory animals, mammals, and humans, lose vitality with age; however, the general level of fundamental knowledge in the biology of lifespan is still at the level of the 1960s of the last century. This position is in sharp contrast to the study of heredity, including modern trends in molecular genetics, although Mendel’s laws were formulated only 50 years after Gompertz’s law [13]. This is due to a number of reasons, including the fact that the object of study, i.e., population data on survival, mortality, and life span, arises after the disappearance of the material substrate. Theoretically, it is possible to identify relationships between negative impacts at the early stages of ontogenesis and their long-term effects on the level of reproductive ability and survival indicators in individual individuals, but such work, due to the large expenditure of time and the difficulty of marking objects, has become possible only at very recently.

The homeostatic ability, expressed in energy units (the ratio of total oxygen consumption to the difference between atmospheric pressure and oxygen tension in mitochondria), which determines reproductive ability, decreases exponentially with age in insects and animals [21]. The same dynamics are revealed by resistance to mastitis in cows. In humans, the wound healing index decreases exponentially, while the frequency of cerebral hemorrhages, aortic aneurysms, and atherosclerosis, on the contrary, increases at the same rate. The decrease in basal metabolism in all animals occurs exponentially, i.e., the rate of decline is higher at a young age.

These experimental data, in principle, are sufficient to clearly separate the concepts of aging and old age; signs of old age are observed in the long term, but the rate of aging (decrease in adaptive capacity, vitality, reserve of protective forces) is maximum at an earlier age, approximately reaching the age of reproductive maturity.

The ontogenetic model explains why it is necessary to look for ways to prevent (and treat) not each individual disease but to act on them as a single group. That is, it is necessary first of all to treat not the final manifestations of diseases, but, as far as possible, to eliminate permanent pathogenetic factors. If health is considered as a dynamic balance of pathogenic and sanogenic factors, then primary health is a combination of innate predisposition to diseases with an innate reserve of sanogenic processes. Health before birth should be considered primary, i.e., obtained with the genome and implemented in antenatal ontogenesis (in the embryonic, transitional, and fetal periods) [26,27,28]. In this regard, it is worth mentioning the 2011 Nobel Prize awarded for work on the discovery of innate immunity resistance. In order to effectively prevent the aging process in humans, clinicians recommend including middle-aged and earlier age periods in the circle of patients. On the same basis, it is recommended to test geroprotectors not on old, but on relatively young individuals, because they have a higher aging rate, and it is advisable to develop adaptogenic drugs for old individuals (at a low aging rate, it is more important to maintain homeostatic ability).

The life span of modern high-yielding productive cows does not reach even half of the period formed in the process of long-term evolution, and the reasons for early culling lie in the discrepancy between the intensity of biosynthetic processes occurring in the udder at the peak of lactation and the metabolic capabilities of internal organs (primarily the liver). This discrepancy, which gives rise to negative shifts in the humoral and immunological status in the mother’s body, inevitably has a negative impact on all stages of prenatal life, including oocyte maturation, ejaculation, and subsequent stages of the embryonic and fetal periods.

As a whole, according to modern views and observational data, the formation of this potential critically depends on the conditions of embryonic development [29,30,31,32,33,34,35,36,37,38], which are formed in cows in the first months of lactation. Therefore, in order to increase the vitality of cows, methods should be found to increase the level of this “primal” health [39,40,41,42,43,44,45].

The results of numerous embryological studies show that in the process of prenatal development, “setting points” for physiological and metabolic processes in adults are determined. Changes in nutritional status during pregnancy can cause adaptive changes in developmental processes due to hormonal shifts in the embryo and fetus, which shift the position of the set points. These adaptive shifts can create short-term positive effects on the embryo and fetus so that the newborn is better prepared for adverse environmental conditions (e.g., undernutrition). However, attempts made in the postnatal period to compensate for growth retardation through increased nutrition can cause metabolic conflicts that predispose to physiological abnormalities in adulthood that increase the risk of diseases [46,47].

Numerous data indicate that metabolic disorders in adulthood often arise as a result of embryological programming of key endocrine systems due to deviations from normal conditions in utero. Inadequate nutrition, ambient temperature, oxygen deficiency, and excess nutrition during pregnancy significantly affect the processes of prenatal development. This means that the optimization of the productive qualities, fertility, general health, welfare, and length of productive life of dairy cows is possible only by taking into account the influence of external environmental factors on the processes of prenatal development.

The first half of lactation in highly productive cows is characterized by the development of energy deficiency, hypoglycemia, massive mobilization of fat depots, accumulation of toxic products that “open the way” for a complex of multifactorial deviations in maternal health, and negative epigenetic modifications in the processes of embryonic development. To decipher the complex picture of metabolic changes occurring during this period, it will be necessary to conduct long-term studies, although certain information can be obtained at the present time by indirect signs according to the data of registration of lactation, indicators of milk composition during automated milking, and the body condition score, which is currently being identified using technical vision systems. At the same time, with the help of such systems, it is possible to register other measurements, including the width and height of the breast, for an indirect assessment of the size of the liver. According to the authors’ hypothesis, the combination of a large increase in udder volume in crossbred animals with a disproportionately smaller increase in liver size may be a causal factor in the development of serious metabolic dysfunctions, including fat hepatosis, decrease in immunity, and, as a consequence, increased culling of cows for a sum of reasons. The adequacy of this hypothesis can be tested on a large amount of empirical data already at the first stages of mastering this new technology.

The discovery of the phenomenon of early prenatal programming of health and diseases, in relation to the problem of the viability of dairy cows, makes us turn to the issues of continuous physiological monitoring of a dairy herd based on modern biosensor systems and Big Data technologies [48,49,50,51]. In the future, as data accumulate, it will be possible to move from health and welfare monitoring to accurate phenotyping and the creation of dairy cattle populations with a balanced combination of productivity and functional reserves of visceral systems [52,53,54,55].

In combination with the improved breeding systems, this scientific and technological complex will provide the necessary basis to expand the list of physiologically justified breeding indices, to reduce morbidity and obtain animals of a new type with indicators of productivity and viability, balanced by a set of economic criteria and biological requirements for milk yield and milk product quality.

## 5. Conclusions

The reason for the antagonism between potential milk yield and productive life span in dairy cow populations is due to the lack of quantitative tests to assess and predict the viability of animals. Multiparametric survival models used in demography and gerontology are practically not applicable to dairy cows for a number of reasons, including an insufficient number of points (number of lactations), population heterogeneity, and others. In our study, a novel methodology was developed to assess survival parameters using data on the number of lactating cows for successive lactations (transverse method) and conduct numerical experiments in silico to take into account the population’s heterogeneity effects using numerical integration in Microsoft Excel.

The conducted research showed that the average productive life span in the studied population of Black-and-White and Khalmogor cows depends mainly on the potential of viability formed in the periods preceding the onset of lactation activity. Taking into account the results of the contemporary research in the field of developmental biology, it can be assumed that the structural prerequisites for this potential are laid down in the processes of embryonic development and in the early periods of postnatal life.

To prevent unfavorable deviations in these processes, it is advisable to carry out continuous monitoring of the physiological status of mothers and young animals and conduct periodic examinations with the registration of possible risk factors. For this, it is necessary to introduce electronic systems of animal identification, devices for medical introscopy, and sets of microdevices for computer analysis of visual and measurement information.

## Figures and Tables

**Table 1:** Age composition of the dairy herd (Tosnensky district of Leningrad oblast).

Years	n	For Successive Lactations, %						
		1	2	3	4–5	6–7	8–9	10 and More
1985	20,528	28.4	21.6	17.0	21.1	8.4	2.8	0.7
1986	20,747	27.4	21.8	17.5	20.7	9.3	2.7	0.6
1987	20,897	28.6	20.9	17.1	21.6	8.7	2.6	0.5
1988	20,596	28.2	20.8	17.2	22.3	8.4	2.6	0.5
Mean %		28.15	21.28	17.20	21.7	8.7	2.68	0.57
Mean n	20,692	5825	4402	3559	4483	1800	554	118

**Table 2:** Analysis of empirical data* for PLS forecasting using the Gompertz function.

Analysis of Empirical Data						Numerical Integration of Gompertz Equation			
t*i*	Ni	ΔNi	*y_i_* = ΔNi/Ni	Δ*n_i_* = ΔNi/N1	Δ*n_i_*×*_i_*	t*i*	*y_i_*	1 − *y_i_*	Ni
1	5825	1423	0.244	0.244	0.24	1	0.244	0.7558	1500
2	4402	843	0.192	0.145	0.29	2	0.192	0.8085	1213
3	3559	922	0.259	0.158	0.47	3	0.259	0.7410	899
4	2637	921	0.349	0.158	0.63	4	0.349	0.6506	585
5	1716	625	0.364	0.107	0.54	5	0.364	0.6359	372
6	1091	416	0.381	0.071	0.43	6	0.381	0.6188	230
7	675	320	0.474	0.055	0.38	7	0.474	0.5256	121
8	355	170	0.480	0.029	0.23	8	0.480	0.5200	63
9	185	97	0.526	0.017	0.15	9	0.526	0.4735	30
10	87	57	0.650	0.010	0.10	10	0.650	0.3500	10
11	31				Σ = 3.5				

* Tosnensky district of Leningrad oblast, 1985–1988. ΣΔ*n_i_*×*_i_* is the average value of PLS.

**Table 3:** Input data for four model populations (MP) *.

Nr	Parameters of MP		Parameter *B* for Subpopulations *N*1 (Initial Number of Cows in Cohort)		
	*B*	*c*			
1	0.1	0.15	0.1		
			1000		
2	0.15	0.13	0.15		0.09
			600		400
3	0.19	0.11	0.22	0.15	0.1
			300	300	400
4	0.23	0.087	0.26	0.20	0.11
			300	300	400

*All IV model populations have the same value of *c* = 0.15; I is homogenous, II consists of two, III and IV consist of three subpopulations, with different values of *B* and initial number of cows in cohort *N*1.

## Data Availability

The data that support the findings of this study are available from the corresponding author upon reasonable request.
